# Risk assessment and prevention of cancer-associated venous thromboembolism in ambulatory patients with solid malignancies

**DOI:** 10.1016/j.rpth.2024.102664

**Published:** 2024-12-24

**Authors:** Nikola Vladic, Cornelia Englisch, Cihan Ay, Ingrid Pabinger

**Affiliations:** Division of Hematology and Hemostaseology, Department of Medicine I, Medical University of Vienna, Vienna, Austria

**Keywords:** cancer, cancer-associated thrombosis, primary prophylaxis, risk assessment, venous thromboembolism

## Abstract

Venous thromboembolism remains a major cause of morbidity and mortality among ambulatory cancer patients, necessitating effective risk assessment and prevention strategies. Despite the availability of risk assessment models and guidelines recommending primary thromboprophylaxis with low-molecular-weight heparins or direct oral anticoagulants, the application of these strategies is inconsistent. This review provides an overview of the current state-of-the-art venous thromboembolism risk assessment and thromboprophylaxis in ambulatory patients with cancer, focusing on existing risk assessment models and the latest guideline recommendations. Finally, it summarizes gaps in knowledge, discusses future directions, and highlights recent advances and state-of-the-art research presented at the 2024 International Society on Thrombosis and Haemostasis Congress in Bangkok, Thailand.

## 1 Introduction

Venous thromboembolism (VTE) remains a critical concern in patients with cancer, contributing to mortality and morbidity [[1], [2], [3], [4]]. VTE in patients with cancer causes a substantial burden, which underlines the importance of its prevention. Patients with cancer who suffer a VTE report a worsened quality of life [5,6], increased psychological burden [4,7], and a shorter overall survival [3,8]. Furthermore, the risk for recurrent VTE and postthrombotic syndrome is increased [9,10], and the treatment with anticoagulation is associated with a higher risk of bleeding compared with the general population [11,12]. Moreover, the need for hospitalization in the acute treatment phase of long-term therapy drives up healthcare costs [13,14].

In this review, we will focus on ambulatory cancer patients, representing a key population for primary prevention strategies based on the concept of precision medicine, which has gained increasing attention in the field of cancer-associated VTE. An important approach to this concept is identifying patients at high risk for VTE based on individual patient characteristics and risk factors. By that, primary prevention can be optimized, ensuring that patients who will likely benefit most are selected. In detail, our State of the Art review aims to provide an overview of current strategies of risk assessment and primary VTE prevention in ambulatory cancer patients while also highlighting novel and relevant research presented during the 2024 International Society on Thrombosis and Haemostasis (ISTH) Congress in Bangkok, Thailand.

## 2 VTE Risk Factors and Risk Assessment

Risk factors contributing to VTE in cancer patients are heterogeneous but can be grouped into patient-, tumor-, and treatment-related factors, as well as predictive biomarkers [2,15].

Among *patient-related factors,* a prior history of VTE and comorbidities are significantly associated with VTE risk [2,16]. *Tumor-related risk factors* (Figure) are important predictors of VTE, with one of the most impactful factors being the tumor site, which is included in almost all major risk assessment models (RAMs) [[17], [18], [19], [20], [21], [22]]. Tumors of the pancreas, stomach, and biliary tract are particularly associated with a high risk of VTE [[23], [24], [25]]. In a Danish population-based study, the highest 6-month cumulative incidence of VTE was found in pancreatic cancer patients [26]. Other tumor-related factors also play a crucial role, as patients with regionally advanced and distant metastatic cancer have been shown to have significantly higher 6-month VTE rates (6.5% and 6.0%, respectively) compared with those with localized disease (2.1%) [27]. Advanced tumor stage, particularly metastatic disease, significantly increases VTE risk and is incorporated into multiple RAMs (Table 1) [21,[29], [30], [31], [32]]. Treatment-related risk factors include the type of systemic anticancer treatment. Chemotherapy is a well-established risk factor for VTE [33], with certain agents, like gemcitabine, platinum-based therapy, and anthracyclines, being incorporated into RAMs [19,29]. As the oncological treatment landscape is constantly evolving, new risk profiles are emerging. In some studies, immune checkpoint inhibitors (ICIs) were associated with the risk of VTE, while conflicting results have been reported in other studies [[34], [35], [36], [37]]. Targeted therapies, such as angiogenesis inhibitors, are associated with VTE risk [38], whereas monotherapy with hormonal agents was recently proposed to be associated with a lower risk [21]. Several *biomarkers* have been linked to increased VTE risk, including D-dimer [39,40], soluble P-selectin [41,42], extracellular vesicle-associated tissue factor activity [43,44], enhanced thrombin generation potential [18,45], and markers of neutrophilic extracellular trap formation, such as citrullinated histone H3 [[46], [47], [48]]. To a different extent, these biomarkers have proven to be useful tools for risk stratification. Furthermore, research is ongoing to determine whether biomarkers can be effectively used for repeated assessments over time to improve risk prediction. This approach could allow for more accurate, longitudinal monitoring of a patient’s risk, offering the potential for personalized and adaptive risk assessments throughout the course of their treatment. In particular, evidence that D-dimer is a promising biomarker for dynamic risk stratification has recently emerged, suggesting that D-dimer increases before the occurrence of a VTE event but remains constant in patients who will not develop VTE [49].

**Figure:**
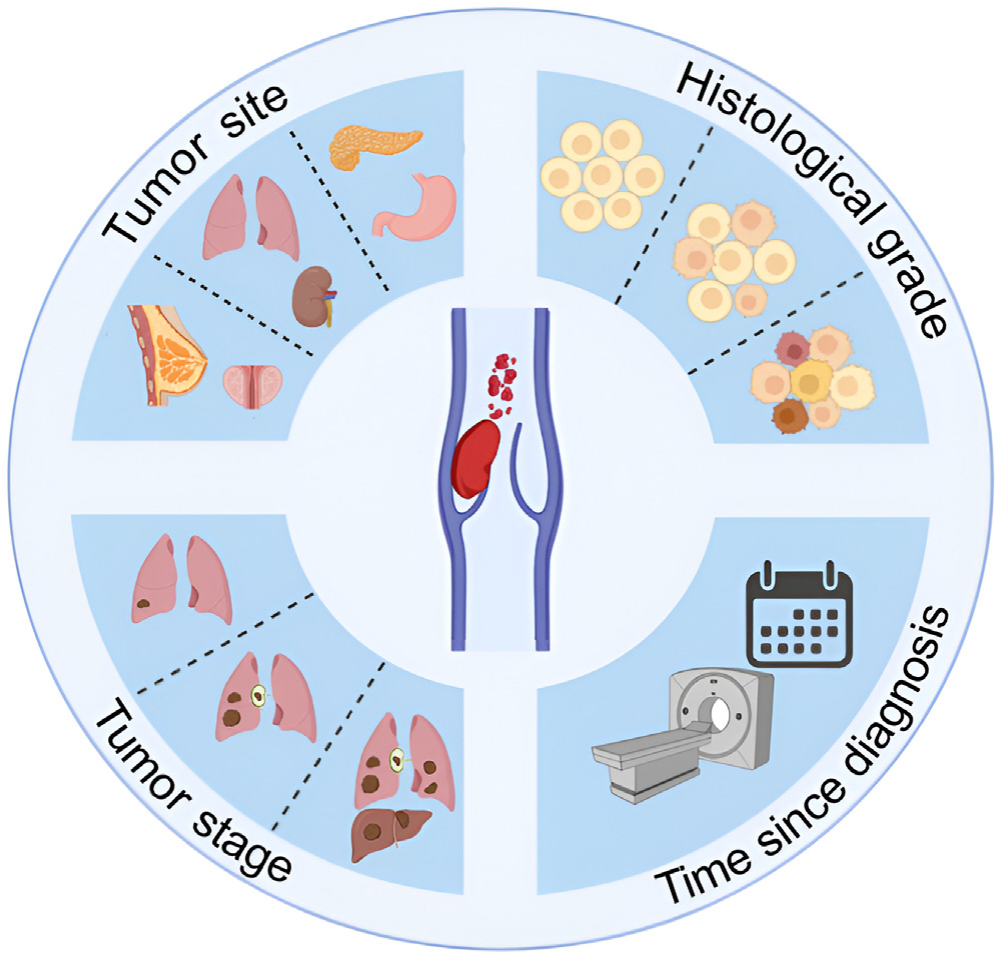
Tumor-related risk factors for cancer-associated venous thromboembolism. Overview of tumor-related risk factors for cancer-associated venous thromboembolism, including histological grade (low to high), time since diagnosis, and tumor stage (progressing from localized disease through lymph node involvement to metastatic disease), and finally, the tumor site-specific risks are represented, with examples of high-risk cancers (pancreas and stomach), intermediate-risk cancers (lung and kidney), and low-risk cancers (breast and prostate). Created with BioRender.com.

**Table 1:** Overview of published risk assessment models for cancer-associated thrombosis.

Parameter	Khorana score^h^ [17]	Vienna modification^i^ [28]	PROTECHT [19]	CONKO [20]	COMPASS-CAT [29]	ONKOTEV [30]	CATScore^j^ [22]	Tic-Onco [31]	ONCO-THROMB [32]	EHR-CAT score^k^ [21]
Very high-risk tumor types^a^	-	-	-	-	-	-	-	-	-	3
High-risk tumor types^b^	2	2	2	2	-	-	Nomogram	X	-	2
Moderate-risk tumor types^c^	1	1	1	1	-	-	Nomogram	X	-	1
Low-risk tumor types^d^	0	0	0	0	-	-	Nomogram	-	-	1
Hb < 10 g/dL or ESA use	1	1	1	1	-	-	-	-	-	1
WBC > 11 G/L	1	1	1	1	-	-	-	-	-	1
Platelets ≥ 350 G/L	1	1	1	1	1	-	-	-	-	1
BMI > 35 kg/m^2^	1	1	1	1	-	-	-	-	X	1
BMI > 25 kg/m^2^	-	-	-	-	-	-	-	X	-	-
Khorana score ≥ 2	-	-	-	-	-	1	-	-	-	-
D-dimer (continuous)	-	-	-	-	-	-	Nomogram	-	-	-
D-dimer ≥ 1.44 μg/L	-	1	-	-	-	-	-	-	-	-
sP-selectin ≥ 53.1 ng/L	-	1	-	-	-	-	-	-	-	-
Gemcitabine therapy	-	-	1	-	-	-	-	-	-	-
Pt-based therapy	-	-	1	-	-	-	-	-	-	-
Targeted and/or endocrine therapy only	-	-	-	-	-	-	-	-	-	−1
Anthracycline therapy	-	-	-	-	6	-	-	-	-	-
Antihormonal therapy in HR+ breast cancer	-	-	-	-	6	-	-	-	-	-
Advanced/metastatic disease	-	-	-	-	2	1	-	-	-	1
Tumor stage	-	-	-	-		-	-	X	X	-
≤6 mo since diagnosis	-	-	-	-	4	-	-	-	-	-
WHO performance status ≥2	-	-	-	1	-	-	-	-	-	-
Prior VTE	-	-	-	-	1	1	-	-	-	1
Vascular/lymphatic compression^e^	-	-	-	-		1	-	-	-	-
Central venous catheter	-	-	-	-	3	-	-	-	-	-
Cardiovascular risk factors^f^	-	-	-	-	5	-	-	-	-	-
Recent hospitalization/acute medical illness^g^	-	-	-	-	5	-	-	-	-	1
Genetic risk score based on SNPs	-	-	-	-	-	-	-	X	9 variants	-
Family history of VTE	-	-	-	-	-	-	-	X	-	-
History of paralysis	-	-	-	-	-	-	-	-	-	1
East/South Asian, Pacific Islander, American Indian or Alaskan Native	-	-	-	-	-	-	-	-	-	−1
**Year of publication**	**2008**	**2010**	**2012**	**2013**	**2017**	**2017**	**2018**	**2018**	**2023**	**2023**
**Number of parameters**	**5**	**7**	**7**	**6**	**9**	**8**	**2**	**6**	**12**	**12**
**External validation**	**Yes**	**Yes**	**Yes**	**Yes**	**Yes**	**Yes**	**Yes**	**Yes**	**Yes**	**Yes**
**Proposed cutoff**	**≥2/≥3**	**Personalized risk prediction**	**≥3**	**≥3**	**≥7**	**Not reported**	**Personalized risk prediction**	**Cutoffs based on sensitivity**	**Cutoffs based on sensitivity**	**≥3**

“X” is used for parameters without a numerical weighting.

BMI, body mass index; ESA, erythropoiesis-stimulating agent; Hb, hemoglobin; HR+, hormone receptor-positive; Pt, platinum; SNP, single nucleotide polymorphism; sP-selectin, soluble P-selectin; VTE, venous thromboembolism; WBC, white blood cell count; WHO, World Health Organization.

a Very high-risk tumor types, according to the EHR-CAT score, include esophageal, gastric, pancreatic, cholangiocarcinoma, and gallbladder.

b High-risk tumor types, according to the EHR-CAT score, include lung, brain, soft tissue sarcoma, kidney, bladder, testicular, uterine, ovarian, multiple myeloma, acute lymphocytic leukemia, precursor B/T lymphoma, other T and natural killer cell lymphoma, diffuse large B cell lymphoma, and Burkitt’s lymphoma; this parameter corresponds to the very high-risk group according to the Khorana score, including pancreatic and gastric cancer.

c Moderate-risk tumor types, according to the EHR-CAT score, include colorectal and intestinal; this parameter corresponds to the high-risk tumor type of the Khorana score, including lung, lymphoma, gynecologic, bladder, and testicular cancer.

d Including all other tumor types not included in the other categories.

e Compression as assessed via magnet resonance imaging.

f Cardiovascular risk factors include 2 or more of the following conditions: peripheral artery disease, ischemic stroke, coronary artery disease, hypertension, hyperlipidemia, diabetes, or obesity.

g Defined as a hospitalization of any length (COMPASS-CAT) or more than 3 days (EHR-CAT) within the last 3 months.

h Risk score calculator available at https://www.mdcalc.com/calc/3315/khorana-risk-score-venous-thromboembolism-cancer-patients

i Risk score calculator available at https://practical-haemostasis.com/Clinical%20Prediction%20Scores/Formulae%20code%20and%20formulae/Formulae/VTED-Cancer/CATS_score.html

j Risk score calculator available at https://catscore.shinyapps.io/catscore/

k Risk score calculator available at https://dynamicapp.shinyapps.io/EHR-CAT/

Among the available RAMs, the Khorana score is the most widely recognized, and it incorporates 5 parameters: cancer site, platelet and leukocyte count, hemoglobin level, and body mass index, and stratifies patients into a low-, intermediate-, or high-risk group for VTE [17]. However, the Khorana score showed varying performances in different external validation studies [17,32,50,51]. To address this, several other RAMs used the Khorana score as a basis and added parameters to enhance risk stratification, such as the Vienna CATScore [52], PROTECHT [19], CONKO [20], and EHR-CAT [21] scores. Despite these advancements, many of these models have failed to show a consistent performance in external validation studies [[53], [54], [55]], highlighting the challenging nature of VTE risk assessment in the cancer patient population. Recently, machine learning (ML) approaches have also been implemented to improve risk prediction by analyzing large datasets and identifying complex patterns that may be difficult to detect via traditional statistical methods. These have led to the development of ML-based RAMs that report promising areas under the curves ranging from 0.66 to 0.83, often outperforming models relying on traditional statistical methods [56]. Nonetheless, the performance gains demonstrated still require external validation to confirm their reliability and reproducibility since ML-based approaches are not without limitations. First of all, they require substantially larger and more varied datasets to capture nuanced relationships accurately, with the rule of thumb for ML models being that the dataset should include at least 10 times as many data points as there are predictor variables [57]. This requirement can be difficult to meet in cancer-associated VTE risk prediction due to the wide range of heterogeneous risk factors present in cancer patients. Additionally, the high number of parameters that need to be assessed for these ML-derived RAMs may limit their use in everyday clinical practice. Given these constraints, it remains to be seen whether ML will offer a definitive improvement in the risk stratification of patients with cancer. Another significant drawback of currently established RAMs is their tendency to dichotomize patients into high- and low-risk groups based solely on a single timepoint assessment. While these categories offer a general estimation of risk, they fail to convey a tangible risk to both the primary oncological provider and the patient. Additionally, this approach fails to appreciate the dynamic nature of VTE risk, which changes over time due to factors such as disease progression [3], the need for hospitalization [58], invasive procedures [59], or changes in treatment (such as initiation of chemotherapy) [33]. Ideally, RAMs should strive to provide a continuous risk percentage (eg, a 6-month risk of VTE) rather than arbitrarily providing a high/low-risk cutoff. For instance, the CATScore estimates the 6-month VTE risk using 2 parameters, tumor site and D-dimer, with the possibility of longitudinal assessment [22].

## 3 Prevention of Cancer-Associated VTE

Despite the advances in identifying risk factors and improving risk prediction of cancer-associated VTE, the prophylactic use of anticoagulation in ambulatory patients with cancer is not routinely applied in clinical practice. This stands in contrast to other clinical scenarios where prophylactic anticoagulation is more consistently used based on risk assessment, such as in atrial fibrillation or the postoperative setting. The 2023 American Heart Association guidelines recommend anticoagulation therapy for males with a CHA₂DS₂-VASc score of 2 or higher and for females with a score of 3 or higher, which corresponds to a 12-month risk of systemic embolism of 2.9% and 4.6%, respectively [60,61]. In the surgical setting, the Caprini score is utilized to assess the risk of VTE. A high Caprini score, defined as 5 or more points, is associated with a 1.8% risk of VTE within 30 days postsurgery and is the recommended cutoff for initiating thromboprophylaxis [62,63]. Notably, cancer patients face an equal or even higher risk of VTE. The 12-month cumulative incidence rate of VTE is estimated to be 3.0% across all cancer types and 5.3% for those receiving chemotherapy or targeted therapy in a recent population-based cohort study [26]. In prospective cohort studies specifically designed to investigate the risk of VTE in patients with cancer, eg, the Vienna Cancer and Thrombosis Study, certain malignancies, such as pancreatic cancer, exhibited higher rates, with a 12-month cumulative incidence of up to 15%. Similarly, patients with gastric and brain cancer show 12-month rates exceeding 10%, similar to findings from other studies [23,64,65].

Current guidelines recommend thromboprophylaxis with either low-molecular-weight heparin (LMWH) or a direct oral anticoagulant (DOAC; apixaban and rivaroxaban) for ambulatory cancer patients at intermediate to high VTE risk, using a validated risk assessment score (Table 2). The 2021 American Society of Hematology guidelines endorse DOACs (apixaban and rivaroxaban) or LMWH for high-risk ambulatory cancer patients and suggest DOACs or no prophylaxis for intermediate-risk patients, as stratified by the Khorana score [68]. The 2023 European Society for Medical Oncology Clinical Practice Guidelines proposes primary thromboprophylaxis (with either LMWH or DOAC) for ambulatory cancer patients facing a high risk, defined as a predicted VTE risk ≥8% to 10% within 6 months after diagnosis, progression, or recurrence of cancer as estimated by a validated risk score [67]. The most recent guidelines have been released this year by the 2024 British Society of Hematology, in which primary thromboprophylaxis is indicated in ambulatory pancreatic cancer patients and may be considered in patients with other cancer sites who are at high risk of VTE using a validated risk assessment score, similar to the 2022 International Initiative on Thrombosis and Cancer guidelines, which also recommends primary thromboprophylaxis for ambulatory pancreatic cancer patients and those with a Khorana score of 2 or more [69,70]. Despite these recommendations, the use of primary thromboprophylaxis in ambulatory cancer patients is inconsistent, likely due to concerns about bleeding risk and the complexities of cancer care. There are increasing efforts being made to increase the awareness and implementation of prophylactic anticoagulation [6].

**Table 2:** Overview of current guidelines for thromboprophylaxis in ambulatory patients with cancer.

Guideline recommendation	ASCO 2020 [66]	ASH 2021 [64]	ITAC 2022 [67]	ESMO 2023 [65]	BSH 2024 [68]
Risk assessment	Khorana score	Khorana score	Khorana score	Validated RAM^b^	Validated RAM^c^
Prophylaxis suggested	≥2	≥1/≥2	Pancreatic cancer^a^ Other cancers: ≥2	6-mo VTE risk ≥8% to 10%	Pancreatic cancer Other cancers: consider assessing with a validated RAM
Low-risk prophylaxis	No prophylaxis	No prophylaxis	No prophylaxis	No prophylaxis	No prophylaxis
Intermediate-risk prophylaxis	**-**	DOAC	**-**	**-**	**-**
High-risk prophylaxis	LMWH or DOAC	LMWH or DOAC	LMWH or DOAC	LMWH or DOAC	LMWH or DOAC^d^

ASCO, American Society of Clinical Oncology; ASH, American Society of Hematology; BSH, British Society for Hematology; DOAC, direct oral anticoagulant; ESMO, European Society for Medical Oncology; ITAC, International Initiative on Thrombosis and Cancer; LMWH, low-molecular-weight heparin; RAM, risk assessment model; VTE, venous thromboembolism.

a Locally advanced or metastatic pancreatic cancer.

b Such as the Khorana score, COMPASS-CAT, or CATScore.

c Any validated RAM.

d The type of anticoagulant should be chosen depending on trial data supporting its efficacy and safety in the patient population in question.

Evidence from randomized controlled trials (RCTs) supports the effectiveness of primary thromboprophylaxis in the ambulatory setting when using established RAMs, as demonstrated in landmark trials like the AVERT (apixaban vs placebo) and CASSINI (rivaroxaban vs placebo) trials [71,72]. A meta-analysis including all major RCTs evaluating primary thromboprophylaxis in patients with cancer reinforced these findings, reporting a relative VTE risk reduction of about 50% with primary prophylaxis, with favorable risk reductions observed for both LMWH and DOACs [73]. A post hoc analysis of the AVERT trial demonstrated that utilizing the CATScore with a 6-month estimated VTE risk cutoff of ≥8% defining high-risk patients resulted in a number needed to treat of just 6 [74]. Finally, a recent RCT, TARGET-TP, illustrates the efficacy of targeted prophylaxis in lung and colorectal cancer patients. By employing a biomarker-based risk stratification approach (D-dimer and fibrinogen), the study demonstrated that the use of thromboprophylaxis reduced the VTE incidence in the high-risk group to that observed in the low-risk group. Furthermore, it was one of the few studies reporting a reduction in all-cause mortality with thromboprophylaxis [75]. However, this trial has several limitations. These include a relatively small sample size (*n* = 328), the inclusion of patients with lung and colorectal cancers only, and the absence of a double-blind setting. Therefore, further RCTs with larger and more diverse cancer cohorts are needed to confirm these findings.

## 4 ISTH Congress Report

At the 2024 ISTH Congress in Bangkok, research on VTE risk assessment and prevention in patients with cancer was presented. To reflect the evolving landscape of anticancer therapies and their impact on VTE, a registry study found that patients receiving chimeric antigen receptor (CAR) T-cell therapy face a significant thrombotic (both venous and arterial) risk, with an observed incidence of 11.6% per person-year [76]. These findings underscore the need to consider potential prophylactic strategies for patients receiving this therapy.

In the area of hematologic malignancy, a prospective side study within an RCT assessed the impact of LMWH in preventing VTE among newly diagnosed acute lymphoblastic leukemia/lymphoma patients [77]. The study found no significant difference in thrombosis incidence between patients who received LMWH (17%) and those who did not (11%) despite a high overall thrombosis rate of 15%. These findings call for an RCT to confirm these results. Finally, a retrospective study of 838 adult patients diagnosed with acute leukemia highlighted the high risk of early VTE, with a 30-day cumulative incidence of 6.6% [78].

The evaluation and validation of RAMs was also a highly researched topic of the presented abstracts at the 2024 ISTH Congress. A study using data from population-based healthcare registries and electronic medical records from the Central Denmark Region, involving 12,471 patients, reported an external validation of the EHR-CAT score [21] and the Khorana score [17] with C-statistics of 0.71 and 0.66, respectively [28]. However, this result contrasted with findings from our research group. An analysis from the prospective Vienna Cancer and Thrombosis and Bleeding (CAT-BLED) study reported a C-statistic of 0.58 for the same 2 RAMs in a cohort of 612 patients [79]. The discrepancy between these results could be attributed to the differences in study design or the higher proportion of patients receiving ICI therapy in the CAT-BLED cohort. Further studies are required to clarify these differences regarding the predictive accuracy of these 2 RAMs. An external validation of the Vienna CATScore [22] within the CAT-BLED study demonstrated its effectiveness in predicting VTE risk in 598 cancer patients across multiple time points (C-statistics, 0.68). By assessing 3 time points and using a 6-month predicted VTE risk threshold of 8% as recommended in current guidelines, the CATScore successfully distinguished between a low- and high-risk group at all time points (6.3% vs 13.6% observed risk, *P* < .001). These results underscore the utility of the CATScore in selecting patients for primary thromboprophylaxis longitudinally during anticancer therapy [80].

## 5 Future Directions

Despite significant advancements in the field of cancer-associated thrombosis, several critical areas require further research and attention. First, the impact of primary thromboprophylaxis on survival in patients with cancer remains unclear. While some studies suggest a potential improvement in survival with anticoagulation, these rarely reported findings remain unconfirmed [75,81,82]. A definitive demonstration that primary thromboprophylaxis not only reduces VTE incidence but also improves prognosis would likely drive greater implementation and acceptance of anticoagulation in this patient population.

Second, the rapidly evolving landscape of oncological therapies also presents new challenges in VTE risk assessment. As cancer treatments advance, leading to improved survival and better prognoses, each new therapeutic modality carries a potential shift in the risk of VTE. This risk may become even more pronounced as cancer patients live longer, shifting the focus toward preventing long-term complications. In particular, further investigation is required to clarify the role of ICIs in VTE risk, as studies have reported conflicting results, and since the first approval in 2011, the implementation of ICI therapy has expanded rapidly [34,35,83]. Regarding CAR T-cell therapy, recent meta-analyses present contrasting findings on VTE rates, with the most recent one indicating that VTE rates in the first 6 months posttreatment are comparable with those seen with chemotherapy, whereas another reports a low incidence of VTE in phase 2 and 3 CAR T-cell therapy trials [84,85]. Antihormonal therapy similarly warrants closer examination. Although tamoxifen is associated with an elevated VTE risk, evidence regarding aromatase inhibitors indicates a lower risk of VTE relative to tamoxifen or chemotherapy [86,87]. Furthermore, a recent study highlights an elevated initial 3-month risk of VTE with antihormonal therapy but a low risk of recurrence thereafter [88]. In targeted therapies, the risk also remains contentious, with epidermal growth factor receptor inhibitors being associated with a high VTE risk, whereas vascular endothelial growth factor inhibitors seem to primarily increase the risk of arterial thromboembolism with inconsistent data regarding VTE risk [[89], [90], [91]]. Moreover, with the advent of novel therapies, including antibody-drug conjugates [92], it is imperative to continuously evaluate the accuracy of existing RAMs in these new therapeutical contexts.

Third, RAMs must be reliable enough to account for these emerging risks while remaining clinically practical. To enhance their utility, future and existing RAMs should ideally minimize the number of required parameters, focus on estimating specific 6-month VTE risk percentages rather than simply categorizing patients into low- or high-risk groups, and allow for reassessment at multiple time points to reflect the dynamic nature of VTE risk in cancer patients. Biomarkers, which have shown promise in capturing the fluctuating risk of VTE, such as D-dimer [39,74], should be further explored for their utility in conjunction with novel anticancer treatments, such as ICIs or antibody-drug conjugates, which may alter VTE risk distinctly different from traditional chemotherapy.

Additionally, the high bleeding risk in cancer patients [11,12,93] has contributed to low implementation rates of primary prophylaxis. Elucidating predictors for the risk of bleeding, such as growth differentiation factor 15 [94] and developing RAMs that integrate both the risk of VTE and bleeding, could significantly improve patient selection, ensuring only those who might benefit most. This would enable more precise risk stratification and targeted primary thromboprophylaxis, ultimately leading to better patient outcomes and more personalized approaches to cancer care.

Lastly, to close existing gaps, the clinical implementation of established RAMs is needed in solid malignancies but also in hematological malignancies, such as lymphoma or acute lymphoblastic leukemia, where the risk of VTE is high but the use of thromboprophylaxis is even less established [95].

## 6 Conclusions

Significant progress has been made in the field of cancer-associated VTE, particularly in the development and refinement of VTE RAMs and the collection of evidence of the efficacy of LMWH and DOACs (apixaban and rivaroxaban) for primary thromboprophylaxis in ambulatory cancer patients. Still, some challenges remain in clinical practice. Despite guideline recommendations, the implementation of primary thromboprophylaxis is insufficient, highlighting the need for increased awareness and adherence to these guidelines. Further research is essential to confirm the potential survival benefits of primary thromboprophylaxis. Additionally, existing RAMs must evolve to accommodate the changing landscape of cancer therapies. Moreover, these models should be simplified for practical use and enable longitudinal risk assessment. Addressing these challenges is crucial for improving outcomes and reducing the burden of VTE in patients with cancer.
